# A systematic review and meta-analysis of the effect of treadmill desks on energy expenditure, sitting time and cardiometabolic health in adults

**DOI:** 10.1186/s12889-021-12094-9

**Published:** 2021-11-13

**Authors:** Akinkunle Oye-Somefun, Zahra Azizi, Chris I. Ardern, Michael A. Rotondi

**Affiliations:** 1 222A Bethune College, York University, 4700 Keele Street, Toronto, ON M3J 1P3 Canada; 2 McGill University Health Centre Research Institute, Centre for Outcomes Research and Evaluation (CORE), 5252 De Maisonneuve Blvd, Montréal, QC, H4A 3S5 Canada; 3 344 Bethune College, York University, 4700 Keele Street, Toronto, ON M3J 1P3 Canada; 4 364 Bethune College, York University, 4700 Keele Street, Toronto, ON M3J 1P3 Canada

**Keywords:** Treadmill, Workstation, Office, Sedentary, Adult, Obesity, meta-analysis

## Abstract

**Background:**

As the health risks of sedentary working environments become more clear, greater emphasis on the implementation of walking interventions to reduce sitting time is needed. In this systematic review and meta-analysis, we investigate the role of treadmill-desk interventions on energy expenditure, sitting time, and cardiometabolic health in adults with sedentary occupations.

**Methods:**

Relevant studies published in English were identified using CINAHL, EMBASE, MEDLINE, Web of Science, Scopus, and PubMed databases up to December 2020. Random effects meta-analysis models were used to pool study results.

**Results:**

Thirteen relevant studies (six workplaces and seven laboratories) were found with a total of 351 participants. Pooled analysis of laboratory studies showed a significant increase in energy expenditure (105.23 kcal per hour, 95% confidence interval [CI]: 90.41 to 120.4), as well as metabolic rate (5.0 mL/kg/min, 95% CI: 3.35 to 6.64), among treadmill desk users compared to sitting conditions. No evidence of significant differences in blood pressure were found. In workplace studies, we observed a significant reduction in sitting time over a 24-h period (− 1.73 min per hour, 95% CI: − 3.3 to − 0.17) among users of treadmill desks, compared to a conventional desk. However, there were no evidence of statistically significant changes in other metabolic outcomes.

**Conclusions:**

Treadmill desks offer a feasible and effective intervention to increase energy expenditure and metabolic rate and reduce sitting time while performing work-related tasks. Future studies are needed to increase generalizability to different workplace settings and further evaluate their impact on cardiometabolic health.

**Supplementary Information:**

The online version contains supplementary material available at 10.1186/s12889-021-12094-9.

## Introduction

Adults spend the majority (i.e., 10 to 13 h) of their waking day being sedentary [1, 2]. High levels of sedentary activity are associated with morbidity and mortality [1–4], including a dose-response relationship between sitting time and mortality from all causes [5, 6] and cardiovascular disease [2, 7]. Thus, the sedentary nature of the work environment supports reconfiguring the workspace to reduce sitting time.

According to the Sedentary Behavior Research Network, sedentary behaviour is defined as energy expenditure (≤ 1.5 metabolic equivalent of task [METs]) that is performed while an individual is awake and not standing [8]. Technological advancements have facilitated more sedentary work environments [9–11] coinciding with fewer opportunities for daily physical activity and energy expenditure in the workplace [12–14]. Moreover, given the impact of the COVID-19 pandemic, working remotely could become routine [15, 16]; thus, targeted efforts will be necessary to address potential decreases in transportation-related energy expenditure and sedentary behaviour that have long-term implications for morbidity and mortality associated with chronic disease [17].

Meanwhile, there is emerging evidence that time spent in light-intensity physical activity, including walking (< 5 mph), can counteract the negative health effects of prolonged sitting [4, 18]. Most recently, there has been growing interest in targeted workplace interventions using active workstations (e.g., sit-stand desks, stepping devices, pedal machines) to interrupt prolonged sitting [19–24] or treadmill desks – a vertical workstation with a treadmill (i.e., a motorized platform with a continuous belt) to permit working and walking [25]. Despite the relative recent emergence of this literature, efforts to understand the effect of a treadmill desk with work-related tasks have, nonetheless, been ongoing since the late 1980s [25]. Several recent studies have explored the use of treadmill desks in the laboratory [26–32], and in the workplace [9, 10, 24, 33–37]; however, there is considerable heterogeneity in intervention characteristics and outcomes, which necessitates analysis by study setting.

To date, few systematic reviews, and meta-analyses of active workstations on sedentary time [38–41], energy expenditure [19, 23, 42], or cardiometabolic health [19, 22, 23, 39] exist. There are even fewer meta-analyses of the effect of treadmill desks on sedentary time [38, 39], energy expenditure [23, 42] and cardiometabolic health [23, 39]. As well, randomized studies [33, 36, 37] and long-term [24, 33] effects of a treadmill desk intervention at work are also rare. Knowing the effect of a treadmill desk both in the laboratory and workplace settings builds on previous works [23, 39–42] and will inform the current discourse on the efficacy and effectiveness of treadmill desks in an increasingly sedentary workplace and work-from-home environment. Thus, the primary objective of this review and meta-analysis is to investigate the effect of a treadmill desk on energy expenditure and sitting time, compared to a conventional desk in sedentary working adults. A secondary objective is to evaluate the potential health effects of using a treadmill desk on cardiometabolic risk factors. We hypothesize that an occupational treadmill desk will be associated with positive changes in energy expenditure, sitting time, and cardiometabolic abnormalities, compared to a conventional desk.

## Methods

Systematic literature searches of CINAHL, EMBASE, MEDLINE, Web of Science, Scopus, and PubMed databases were performed to identify relevant peer-reviewed journal articles, related to treadmill desk use in the workplace up to December 2020. The search terms included (but were not limited to) “sedentary behavior”, “sedentary lifestyle”, “active workstation”, “treadmill workstation”, “treadmill desk”, “walking or physical activity”, “stepping device”, “workplace”, “office” alone or combined using Boolean operators in each database. Full electronic search strategy from PubMed is included in supplementary material (see Table S1). Manual searches were performed using the reference lists of retrieved articles to locate additional articles. There was no language restriction when conducting the search. This study was conducted based on preferred reporting items for systematic reviews and meta-analyses (PRISMA) guidelines for systematic reviews [43]. There was no potential for participant identification from published summary data; thus, ethics approval was not required from our host institution.

### Inclusion criteria

The PRISMA flow diagram [43] in Fig. 1 summarizes the selection of studies. Eligible studies were screened by title and abstract. Potential studies were included if their study designs involved interventions to reduce sitting (e.g., pre-post studies, prospective clinical trials, prospective controlled designs, repeated-measures, randomized repeated-measures, randomized controlled trials, and randomized cross-over trials). Studies were restricted to include healthy adults ages 18+ with a normal to overweight/obese body mass index (BMI, calculated as body mass (kg) divided by the square of the standing height, kg/m^2^) [36].

### Data extraction and assessment of bias

Full-text articles were independently assessed for eligibility by two reviewers (AO-S, ZA) using the Critical Appraisal Skills Programme (CASP) checklists [44]. This was followed by data extraction and assessment of bias using the Cochrane Collaboration tool (i.e., the quality assessment and data extraction form) [45]. Relevant information from individual trials included study design, sample characteristics, intervention modality, duration, frequency of interaction, and primary outcomes of interest. Authors [30, 34, 37] were contacted for additional data where required. The mean differences for each outcome along with their 95% confidence intervals [CI] for intervention versus control groups were extracted using a pre-specified format. Discrepancies in this process were resolved by consensus or by consultation with a third reviewer (CIA).

### Outcomes

The primary study outcomes were changes in objectively monitored energy expenditure and sitting time. The secondary outcomes were physiological changes (i.e., oxygen consumption, blood pressure, fasting glucose, high density lipoprotein, triglycerides, total cholesterol, body fat percentage, and body mass index (BMI)). Energy expenditure was expressed as kilocalories per hour to be consistent with previous research [29] and estimated from the reported average METs and body mass [26]. One MET – the energy cost at rest or sitting quietly – is equivalent to 3.5 ml of oxygen per kilogram of body mass per minute [30]. Similarly, sitting time was expressed as minutes per hour and estimated from the reported total time spent sitting/lying per day [34, 35] or postural time (i.e., the time spent walking, standing, and sitting or resting/lying) during waking or working hours [24, 33].

### Statistical analyses

Mean differences were the principal summary measure for analysis and were obtained by calculating the difference between baseline and follow-up for pre-post studies, or treadmill desk users and control in parallel arm designs. Analysis was stratified by study setting (i.e., laboratory and workplace). Mean differences from individual studies were pooled using the *metafor* package [46] in R [47]. The *I*^2^ statistic was used to assess the heterogeneity between studies [48]. Studies were assumed to be heterogeneous; thus, random-effects were modelled to calculate pooled effect sizes.

## Results

### Search strategy

Figure 1 shows the flow diagram for the inclusion of studies in this report consistent with the PRISMA statement [43]. A total of 13 articles met the criteria for quantitative synthesis including seven studies carried out in a laboratory, and six studies carried out in a workplace setting. As a result, the total number of participants available for meta-analysis was 351, including 117 participants in a laboratory, and 234 in a workplace setting. Laboratory assessments varied between five and 30 min, while workplace assessments at follow-up varied between three and 18 months.

#### Treadmill desk

Included studies assessed the impact of a treadmill desk, compared to a conventional desk. The characteristics of included studies in the laboratory and workplace for the treadmill desk group are presented in additional files (see Additional file 1: Table 1 and Table 2).

### Quantitative synthesis

Eligible studies were pooled for analysis by study setting. Results of the pooled estimates for studies conducted in a laboratory or workplace are presented in additional files (see Additional file 1: Table 3 and Table 4). Forest plots (see Additional file 2: Fig. 2, and Fig. 3), and funnel plots (see Additional file 2: Fig. 4, and Fig. 5) of pooled estimates, are presented in supplementary material.

#### Laboratory settings

The random effects model showed that there was a significant increase in energy expenditure (105.23 kcal per hour, 95% CI: 90.41 to 120.04 kcal per hour; *I*^2^ = 61.22%), as well as a significant increase in the oxygen consumption (5.0 mL/kg/min, 95% CI: 3.35 to 6.64 mL/kg/min; *I*^2^ = 86.19%), as a result of slow walking (≤ 3 mph) on a treadmill desk compared to sitting. We observed no significant change in systolic (− 1.26 mmHg, 95% CI: − 6.85 to 4.33 mmHg; *I*^2^ **=** 45.57%) or diastolic blood pressure (− 1.79 mmHg, − 5.08 to 1.50 mmHg; *I*^2^ **=** 18.46%) in the pooled analysis.

#### Workplace settings

The random effects model revealed a statistically significant reduction in sitting time over a 24-h period (− 1.73 min per hour, 95% CI: − 3.30 to − 0.17, *I*^2^ **=** 42.4%), among users of treadmill desks, compared to a conventional desk. In an exploratory subgroup analysis of only randomized controlled trials [33, 36], we noted similar reductions in sitting time among treadmill desk users compared to the usual condition during working hours, which did not reach statistical significance (− 3.58 min per hour, 95% CI: − 7.17 to 0.02). There were no significant changes in physiological outcomes (blood pressure, glucose, high density lipoproteins, triglycerides, total cholesterol, body fat percentage, and BMI) with treadmill desk use; however, the direction of effect favoured the intervention for most outcomes, with the exception of BMI (Additional file 1: Table 4).

## Discussion

A systematic review and meta-analysis were performed to investigate the effect of a treadmill desk on energy expenditure, sitting time, and cardiometabolic health among adults in laboratory and workplace settings. Previous research has shown that irrespective of the time spent in physical activity, prolonged sedentary activity is associated with negative health effects such as cardiometabolic abnormalities [5, 49–51]. Furthermore, drug therapies to reduce symptoms of cardiometabolic risk factors are not without adverse side effects [52]. With many individuals also working from home [12–16], use of treadmill desks may be a feasible alternative to conventional desks for reducing prolonged sitting and enhancing cardiometabolic health among adults with sedentary occupations.

The findings of this meta-analysis showed some potential health benefits in both the analysis of laboratory, and workplace studies. Notably, we observed a significant increase in energy expenditure (primary outcome) and metabolic rate, as well as potential, though non-significant reductions in blood pressure among users of a treadmill desk in a laboratory setting. In the analysis of workplace studies, we observed a small but clinically relevant reduction in sitting time (primary outcome) during a full-day as a result of treadmill desk interventions. An exploratory analysis of only randomized controlled trials also revealed reductions (non-significant) in sitting time during working hours. Although we observed non-significant physiological changes (i.e., blood pressure, fasting glucose, high density lipoprotein, triglycerides, total cholesterol, body fat percentage, and BMI) in the workplace, the direction of effect typically favoured the intervention, suggesting that it may be a lack of statistical power. Individuals with prediabetes or prehypertension [53] could also benefit from the positive health effects associated with treadmill desks.

To date, a number of biological and physiological factors have been implicated in the pathway between sedentary activity on cardiometabolic health [54], including lipoprotein lipase activity [7], oxidative stress [55, 56], and insulin resistance [57]. Although these mechanisms are not fully understood [17, 58], sedentary behavior is an emerging independent risk factor of cardiometabolic diseases independent of physical activity status [2, 7, 53]. Our systematic review and meta-analysis aligns with the call of the American Heart Association [17] for interventions for reducing sedentary behaviour and its effects.

Our results are consistent with previous meta-analyses of workplace interventions, including treadmill desks, on energy expenditure and sedentary time [23, 38, 39, 42]. However, there was considerable heterogeneity [48] in the included trials. As such, there was a need to look at these collectively to ascertain the overall efficacy and effectiveness of these interventions. Although two workplace studies measured energy expenditure [35, 37], this was insufficient for pooled analysis to ascertain changes in energy expenditure among treadmill desk users. There were minimal issues ascertaining energy expenditure in laboratory studies. However, our findings agree with previous research that the opportunity cost of sitting, instead of walking, amounts to ~ 100 kcal per hour [2, 22]. Although the effect size for sitting time in our study was small, it aligns with prior randomized and observational studies which have shown improvements in insulin sensitivity [59] and mortality risk [60, 61] as a result of a 30-min reduction in sitting time per day; accrued in short (1- to 2-min) bouts at regular intervals.

Importantly, short bouts of light-intensity walking at regular intervals may be easier for sedentary adults to maintain, and it has also been shown to counteract shear stress due to prolonged sitting [62]. Indeed, lower extremity shear stress worsens within one hour of uninterrupted sitting, which promotes superficial femoral artery endothelial dysfunction [62]. However, standing as an activity on its own for counteracting prolonged sitting may also be taxing on the heart. Cyclic contraction of the leg muscles (e.g., walking) coinciding with increased respiration offers more support to the pumping mechanism of the heart [63]. Moreover, walking circulates antioxidants for counteracting oxidative stress or reactive oxygen species [56], and; in animal models, maintenance of daily walking has been shown to increase lipoprotein lipase activity, a key regulator of lipid metabolism [64]. Thus, interrupting prolonged sitting with walking may be more beneficial for weight management, glucose and lipid metabolism, and reduced mortality risk in sedentary adults [65]. Importantly, previous research [21, 66] has found that walking and working has no considerable negative effects on work-related tasks. As a result, our findings reinforce that in the absence of opportunities for moderate intensity walking in many workplace settings [31], long-term sustained light-intensity walking can be achieved with the installation of treadmill desks.

### Strengths and limitations

An important strength of this systematic review and meta-analysis is the inclusion of valid and reliable measures of energy expenditure, and objectively monitored changes in sitting time using accelerometers; thus, detection bias was low. Analysis was stratified according to study setting, and there was also low risk of publication bias. Finally, the inclusion of studies with long-term follow-up and randomization, and the pooling of studies to produce a large sample size allowed for a more comprehensive analysis of these effects.

Nonetheless, there were some limitations. Although this meta-analysis was aimed at the generally sedentary working adult population, there were only a few studies in the literature examining the effect of treadmill desks on sedentary behaviour, and even fewer with proper randomization. Among the studies with appropriate randomization, one study had sit-stand desks [33] as the conventional desk, and one study had shared treadmill desks [36] as the intervention, contrary to other intervention studies. To account for this variation in experimental strategy, sitting time during working hours was explored. Furthermore, studies with randomization did not mask study personnel, with the exception of one workplace study that masked researchers to the group assignments [33], and one laboratory study that masked assessors to the purpose of the study and protocol [28]. The risk of contamination was also a concern as participants in the same workplace were likely to interact socially [20], and behavioural, extraneous, observer, and seasonal effects cannot be ruled out. There were also limitations in objective sedentary time. Notably, waking hours and non-wear time were not necessarily specified, and neither were exposure levels; therefore, we can only speculate about the dose-response relationships [67]. Although there was potential for attrition [24, 33, 36, 37], losses to follow-up were accounted for, resulting in good participant retention.

Treadmill desks offer a feasible approach for facilitating reductions in sitting time in an increasingly sedentary workplace and work-from-home environment. Studies with randomization, longer follow-up times and larger sample sizes are nonetheless necessary to evaluate their impact in real-world workplace settings. On the basis of the above, our findings suggest that future studies should involve: i) cluster randomized controlled trials in order to compare the effects of treadmill desks to reduce sitting time across workplaces, and to reduce the risk of treatment group contamination; ii) experimental designs that include the investigation of dose-response relationships to support the efficacy and effectiveness of treadmill desks, given the potential for the intervention to have positive health effects; iii) 24-h ambulatory blood pressure monitors or wrist-worn devices that may reduce the discomfort of cuffs and interruptions in sleep, and accelerometers for 24-h movement patterns to account for non-wear time and waking hours, in order to ascertain work and non-work sedentary time, and; iv) behavioural support strategies such as health promotion programs [24], automated reminders [33], diarizing physical activity [34], and exercise counselling [36, 37] to improve adherence. Indeed, our study further highlights the need for integrated workplace interventions, as interventions that combine activity monitors and health education are thought to be more effective at reducing sedentary behaviours [68, 69].

## Conclusion

Our study shows that the use of treadmill desks increases energy expenditure and metabolic rate and reduces sitting time. Traditional levels of significance were not observed for any of the physiological markers in the workplace setting; however, point estimates show that there is potential for improvements in cardiometabolic health. These findings suggest that more studies are needed, as treadmill desks may counteract the negative health effects of prolonged sitting in the working environment, but the current evidence is inconclusive. Future studies with longer-term follow-up and larger sample sizes are needed to better understand the effects of treadmill desks on sitting time and various cardiometabolic health parameters, particularly as it relates to free-living populations. To this end, well-crafted cluster randomized controlled trials would further determine the effectiveness of these interventions.

## Supplementary Information

**Additional file 1: Table 1.** Characteristics of the included studies in the laboratory settings. Description of data: study design, outcome of interest, estimated mean, and mean difference. **Table 2.** Characteristics of the included studies in the workplace settings. Description of data: study design, outcome of interest, estimated mean, and mean difference. **Table 3.** Treadmill desk in laboratory setting. Description of data: Effect estimates. **Table 4.** Treadmill desk in workplace setting. Description of data: Effect estimates

**Additional file 2: Figure 1.** Flowchart of studies included in the meta-analysis. Description of data: Flowchart of studies included in the meta-analysis. **Figure 2.** Forest plots of studies in laboratory settings. Description of data: Outcomes are as follows: (**a**) energy expenditure; (**b**) rate of oxygen consumption; (**c**) systolic blood pressure; (**d**) diastolic blood pressure. Error bars represent the 95% Confidence Interval. **Data source:** Botter et al., 2016 [26]; Champion et al., 2018 [27]; Cox et al., 2011 [28]; Levine & Miller, 2007 [29]; Schuna et al., 2019 [30]; Zeigler et al., 2015 [31]; Zeigler et al., 2016 [32]. **Figure 3.** Forest plots of studies in workplace settings. Description of data: Outcomes are as follows: (**a**) sitting time (minutes per hour) during a full-day; (**b**) sitting time (minutes per hour) during working hours; (**c**) systolic blood pressure; (**d**) diastolic blood pressure; (**e**) glucose concentrations; (**f**) high density lipoproteins: (**g**) triglycerides; (**h**) total cholesterol; (**i**) body fat percentage; (**j**) BMI. Error bars represent the 95% Confidence Interval. **Data source:** Wahlström et al., 2019 [24]; Bergman et al., 2018 [33]; John et al., 2011 [34]; Koepp et al., 2013 [35]; Schuna et al., 2014 [36]; Thompson et al., 2014 [37]. **Figure 4.** Funnel plots of studies in laboratory settings. Description of data: Outcomes are as follows: (**a**) energy expenditure; (**b**) rate of oxygen consumption; (**c**) systolic blood pressure; (**d**) diastolic blood pressure. Error bars represent the 95% Confidence Interval. **Data source:** Botter et al., 2016 [26]; Champion et al., 2018 [27]; Cox et al., 2011 [28]; Levine & Miller, 2007 [29]; Schuna et al., 2019 [30]; Zeigler et al., 2015 [31]; Zeigler et al., 2016 [32]. **Figure 5.** Funnel plots of studies in workplace settings. Description of data: Outcomes are as follows: (**a**) sitting time (minutes per hour) during a full-day; (**b**) sitting time (minutes per hour) during working hours; (**c**) systolic blood pressure; (**d**) diastolic blood pressure; (**e**) glucose concentrations; (**f**) high density lipoproteins: (**g**) triglycerides; (**h**) total cholesterol; (**i**) body fat percentage; (**j**) BMI. Error bars represent the 95% Confidence Interval. **Data source:** Wahlström et al., 2019 [24]; Bergman et al., 2018 [33]; John et al., 2011 [34]; Koepp et al., 2013 [35]; Schuna et al., 2014 [36]; Thompson et al., 2014 [37]

**Additional file 3: Table S1.** Electronic search strategy

## Data Availability

All data analysed during this study are available from the published articles (and their supplementary information files) or from their corresponding authors on reasonable request.

## Abbreviations

MET: metabolic equivalent of task
CINAHL: cumulative index of nursing and allied health literature
EMBASE: *excerpta medica* database
MEDLINE: medical literature analysis and retrieval system online
PRISMA: preferred reporting items for systematic reviews and meta-analyses
RCT: randomized controlled trial
CASP: Critical Appraisal Skills Programme
BMI: body mass index
CI: confidence intervals
kcal: kilocalorie
mph: miles per hour
kmph: kilometres per hour
BP: blood pressure
FG: fasting glucose
HDL: high density lipoprotein
TG: triglycerides
TC: total cholesterol
BF: body fat

## References

[1] Inoue M, Iso H, Yamamoto S, Kurahashi N, Iwasaki M, Sasazuki S, Tsugane S, Japan Public Health Center-Based Prospective Study Group (2008). Daily Total physical activity level and premature death in men and women: results from a large-scale population-based cohort study in Japan (JPHC study). Ann Epidemiol.

[2] Pandey A, Salahuddin U, Garg S, Ayers C, Kulinski J, Anand V, Mayo H, Kumbhani DJ, de Lemos J, Berry JD (2016). Continuous dose-response association between sedentary time and risk for cardiovascular disease: a Meta-analysis. JAMA Cardiol.

[3] Thorp AA, Owen N, Neuhaus M, Dunstan DW (2011). Sedentary behaviors and subsequent health outcomes in adults a systematic review of longitudinal studies, 1996-2011. Am J Prev Med.

[4] Dunstan DW, Howard B, Healy GN, Owen N (2012). Too much sitting – a health hazard. Diabetes Res Clin Pract.

[5] Löllgen H, Böckenhoff A, Knapp G (2009). Physical activity and all-cause mortality: an updated meta-analysis with different intensity categories. Int J Sports Med.

[6] Matthews CE, Keadle SK, Troiano RP, Kahle L, Koster A, Brychta R, van Domelen D, Caserotti P, Chen KY, Harris TB, Berrigan D (2016). Accelerometer-measured dose-response for physical activity, sedentary time, and mortality in US adults. Am J Clin Nutr.

[7] Katzmarzyk PT, Church TS, Craig CL, Bouchard C (2009). Sitting time and mortality from all causes, cardiovascular disease, and Cancer. Med Sci Sports Exerc.

[8] Tremblay MS, Aubert S, Barnes JD, Saunders TJ, Carson V, Latimer-Cheung AE (2017). Sedentary behavior research network (SBRN) – terminology consensus project process and outcome. Int J Behav Nutr Phys Act.

[9] Ben-Ner A, Hamann DJ, Koepp G, Manohar CU, Levine J (2014). Treadmill workstations: the effects of walking while working on physical activity and work performance. PLoS One.

[10] Parry S, Straker L, Gilson ND, Smith AJ (2013). Participatory workplace interventions can reduce sedentary time for office workers—a randomised controlled trial. PLoS One.

[11] Ryan CG, Dall PM, Granat MH, Grant PM (2011). Sitting patterns at work: objective measurement of adherence to current recommendations. Ergonomics.

[12] Cucinotta D, Vanelli M (2020). WHO declares COVID-19 a pandemic. Acta Biomed.

[13] Cowling BJ, Aiello AE (2020). Public health measures to slow community spread of coronavirus disease 2019. J Infect Dis.

[14] Stockwell S, Trott M, Tully M, Shin J, Barnett Y, Butler L, McDermott D, Schuch F, Smith L (2021). Changes in physical activity and sedentary behaviours from before to during the COVID-19 pandemic lockdown: a systematic review. BMJ Open Sport Exerc Med.

[15] Gallo LA, Gallo TF, Young SL, Moritz KM, Akison LK (2020). The impact of isolation measures due to COVID-19 on energy intake and physical activity levels in Australian University students. Nutrients.

[16] Budd J, Miller BS, Manning EM, Lampos V, Zhuang M, Edelstein M, Rees G, Emery VC, Stevens MM, Keegan N, Short MJ, Pillay D, Manley E, Cox IJ, Heymann D, Johnson AM, McKendry RA (2020). Digital technologies in the public-health response to COVID-19. Nat Med.

[17] Young DR, Hivert M-F, Alhassan S, Camhi SM, Ferguson JF, Katzmarzyk PT, Lewis CE, Owen N, Perry CK, Siddique J, Yong CM, Physical Activity Committee of the Council on Lifestyle and Cardiometabolic Health; Council on Clinical Cardiology; Council on Epidemiology and Prevention; Council on Functional Genomics and Translational Biology; and Stroke Council (2016). Sedentary behavior and cardiovascular morbidity and mortality: a science advisory from the American Heart Association. Circulation.

[18] Duvivier BMFM, Schaper NC, Bremers MA, van Crombrugge G, Menheere PPCA, Kars M, Savelberg HHCM (2013). Minimal intensity physical activity (standing and walking) of longer duration improves insulin action and plasma lipids more than shorter periods of moderate to vigorous exercise (cycling) in sedentary subjects when energy expenditure is comparable. PLoS One.

[19] Dupont F, Léger P-M, Begon M, Lecot F, Sénécal S, Labonté-Lemoyne E, Mathieu ME (2019). Health and productivity at work: which active workstation for which benefits: a systematic review. Occup Environ Med.

[20] Holtermann A (2018). Treadmill workstations versus sit–stand desks for increasing physical activity. Lancet Public Health.

[21] Larson MJ, LeCheminant JD, Carbine K, Hill KR, Christenson E, Masterson T (2015). Slow walking on a treadmill desk does not negatively affect executive abilities: an examination of cognitive control, conflict adaptation, response inhibition, and post-error slowing. Front Psychol.

[22] MacEwen BT, MacDonald DJ, Burr JF (2015). A systematic review of standing and treadmill desks in the workplace. Prev Med.

[23] Podrekar N, Kozinc Ž, Šarabon N. Effects of cycle and treadmill desks on energy expenditure and cardiometabolic parameters in sedentary workers: review and meta-analysis. Int J Occup Saf Ergon. 2021;27(3):728-36. 10.1080/10803548.2018.1562688. 10.1080/10803548.2018.1562688 30595127

[24] Wahlström V, Bergman F, Öhberg F, Eskilsson T, Olsson T, Järvholm LS (2019). Effects of a multicomponent physical activity promoting program on sedentary behavior, physical activity and body measures: a longitudinal study in different office types. Scand J Work Environ Health.

[25] Edelson N, Danoffz J (1989). Walking on an electric treadmill while performing VDT office work. SIGCHI Bull.

[26] Botter J, Ellegast RP, Burford E-M, Weber B, Könemann R, Commissaris DACM (2016). Comparison of the postural and physiological effects of two dynamic workstations to conventional sitting and standing workstations. Ergonomics.

[27] Champion RB, Smith LR, Smith J, Hirlav B, Maylor BD, White SL, Bailey DP (2018). Reducing prolonged sedentary time using a treadmill desk acutely improves cardiometabolic risk markers in male and female adults. J Sports Sci.

[28] Cox RH, Guth J, Siekemeyer L, Kellems B, Brehm SB, Ohlinger CM (2011). Metabolic cost and speech quality while using an active workstation. J Phys Act Health.

[29] Levine JA, Miller JM (2007). The energy expenditure of using a “walk-and-work” desk for office workers with obesity. Br J Sports Med.

[30] Schuna JM, Hsia DS, Tudor-Locke C, Johannsen NM (2019). Energy expenditure while using workstation alternatives at self-selected intensities. J Phys Act Health.

[31] Zeigler ZS, Swan PD, Bhammar DM, Gaesser GA (2015). Walking workstation use reduces ambulatory blood pressure in adults with prehypertension. J Phys Act Health.

[32] Zeigler ZS, Mullane SL, Crespo NC, Buman MP, Gaesser GA (2016). Effects of standing and light-intensity activity on ambulatory blood pressure. Med Sci Sports Exerc.

[33] Bergman F, Wahlström V, Stomby A, Otten J, Lanthén E, Renklint R, Waling M, Sörlin A, Boraxbekk CJ, Wennberg P, Öhberg F, Levine JA, Olsson T (2018). Treadmill workstations in office workers who are overweight or obese: a randomised controlled trial. Lancet Public Health.

[34] John D, Thompson DL, Raynor H, Bielak K, Rider B, Bassett DR (2011). Treadmill workstations: a worksite physical activity intervention in overweight and obese office workers. J Phys Act Health.

[35] Koepp GA, Manohar CU, McCrady-Spitzer SK, Ben-Ner A, Hamann DJ, Runge CF (2013). Treadmill desks: a 1-year prospective trial. Obesity.

[36] Schuna JM, Swift DL, Hendrick CA, Duet MT, Johnson WD, Martin CK, Church TS, Tudor-Locke C (2014). Evaluation of a workplace treadmill desk intervention: a randomized controlled trial. J Occup Environ Med.

[37] Thompson WG, Koepp GA, Levine JA (2014). Increasing physician activity with treadmill desks. Work.

[38] Chu AHY, Ng SHX, Tan CS, Win AM, Koh D, Müller-Riemenschneider F (2016). A systematic review and meta-analysis of workplace intervention strategies to reduce sedentary time in white-collar workers. Obes Rev.

[39] Neuhaus M, Eakin EG, Straker L, Owen N, Dunstan DW, Reid N, Healy GN (2014). Reducing occupational sedentary time: a systematic review and meta-analysis of evidence on activity-permissive workstations. Obes Rev.

[40] Shrestha N, Kukkonen-Harjula KT, Verbeek JH, Ijaz S, Hermans V, Pedisic Z (2018). Workplace interventions for reducing sitting at work. Cochrane Database Syst Rev.

[41] Blackburn NE, Wilson JJ, McMullan II, Caserotti P, Giné-Garriga M, Wirth K (2020). The effectiveness and complexity of interventions targeting sedentary behaviour across the lifespan: a systematic review and meta-analysis. Int J Behav Nutr Phys Act.

[42] Cao C, Liu Y, Zhu W, Ma J (2016). Effect of active workstation on energy expenditure and job performance: a systematic review and Meta-analysis. J Phys Act Health.

[43] Moher D, Liberati A, Tetzlaff J, Altman DG (2009). The PRISMA group. Preferred Reporting Items for Systematic Reviews and Meta-Analyses: The PRISMA Statement BMJ.

[44] Critical Appraisal Skills Programme. CASP (systematic review) checklist. 2018. Available at: https://casp-uk.net/casp-tools-checklists.

[45] Higgins JPT, Thomas J, Chandler J, Cumpston M, Li T, Page MJ, et al. Cochrane Handbook for Systematic Reviews of Interventions version 6.1: Cochrane; 2020. Available at: https://training.cochrane.org/handbook.

[46] Viechtbauer W. Metafor: Meta-analysis package for R (Version 2.4–0). 2020. Available at: https://cran.r-project.org/web/packages/metafor/index.html.

[47] R Core Team. R: A language and environment for statistical computing. Vienna, Austria: R Foundation for Statistical Computing; 2020. Available at: http://www.R-project.org/.

[48] Higgins JPT, Thompson SG (2002). Quantifying heterogeneity in a meta-analysis. Stat Med.

[49] Beunza JJ, Martínez-González MÁ, Ebrahim S, Bes-Rastrollo M, Núñez J, Martínez JA, Alonso A (2007). Sedentary behaviors and the risk of incident HypertensionThe SUN cohort. Am J Hypertens.

[50] Ford ES, Kohl HW, Mokdad AH, Ajani UA (2005). Sedentary behavior, physical activity, and the metabolic syndrome among U.S. adults. Obes Res.

[51] Thorp AA, Healy GN, Owen N, Salmon J, Ball K, Shaw JE, Zimmet PZ, Dunstan DW (2010). Deleterious associations of sitting time and television viewing time with Cardiometabolic risk biomarkers: Australian diabetes, obesity and lifestyle (AusDiab) study 2004–2005. Diabetes Care.

[52] Fredman G, Ozcan L, Tabas I (2014). Common Therapeutic Targets in Cardiometabolic Disease. Sci Transl Med.

[53] Healy GN, Winkler EAH, Eakin EG, Owen N, Lamontagne AD, Moodie M (2017). A Cluster RCT to Reduce Workers’ Sitting Time: Impact on Cardiometabolic Biomarkers. Med Sci Sports Exerc.

[54] Lavie CJ, Ozemek C, Carbone S, Katzmarzyk PT, Blair SN (2019). Sedentary behavior, exercise, and cardiovascular health. Circ Res.

[55] Lessiani G, Santilli F, Boccatonda A, Iodice P, Liani R, Tripaldi R, Saggini R, Davì G (2016). Arterial stiffness and sedentary lifestyle: role of oxidative stress. Vasc Pharmacol.

[56] Gardner AW, Montgomery PS, Zhao YD, Silva-Palacios F, Ungvari Z, Csiszar A, Sonntag WE (2017). Association between daily walking and antioxidant capacity in patients with symptomatic peripheral artery disease. J Vasc Surg.

[57] Qi Q, Strizich G, Merchant G, Sotres-Alvarez D, Buelna C, Castañeda SF, Gallo LC, Cai J, Gellman MD, Isasi CR, Moncrieft AE, Sanchez-Johnsen L, Schneiderman N, Kaplan RC (2015). Objectively measured sedentary time and Cardiometabolic biomarkers in US Hispanic/Latino adults: the Hispanic community health study/study of Latinos (HCHS/SOL). Circulation.

[58] Haskell WL, Lee IM, Pate RR, Powell KE, Blair SN, Franklin BA (2007). Physical activity and public health: updated recommendation for adults from the American College of Sports Medicine and the American Heart Association. Med Sci Sports Exerc.

[59] Dunstan DW, Kingwell BA, Larsen R, Healy GN, Cerin E, Hamilton MT, Shaw JE, Bertovic DA, Zimmet PZ, Salmon J, Owen N (2012). Breaking up prolonged sitting reduces postprandial glucose and insulin responses. Diabetes Care.

[60] Beddhu S, Wei G, Marcus RL, Chonchol M, Greene T (2015). Light-intensity physical activities and mortality in the United States general population and CKD subpopulation. Clin J Am Soc Nephrol.

[61] Diaz KM, Duran AT, Colabianchi N, Judd SE, Howard VJ, Hooker SP (2019). Potential effects on mortality of replacing sedentary time with short sedentary bouts or physical activity: a National Cohort Study. Am J Epidemiol.

[62] Thosar SS, Bielko SL, Mather KJ, Johnston JD, Wallace JP (2015). Effect of prolonged sitting and breaks in sitting time on endothelial function. Med Sci Sports Exerc.

[63] Secomb TW (2016). Hemodynamics. Compr Physiol.

[64] Bey L, Hamilton MT (2003). Suppression of skeletal muscle lipoprotein lipase activity during physical inactivity: a molecular reason to maintain daily low-intensity activity. J Physiol.

[65] Füzéki E, Engeroff T, Banzer W (2017). Health benefits of light-intensity physical activity: a systematic review of accelerometer data of the National Health and nutrition examination survey (NHANES). Sports Med.

[66] Fidler JL, MacCarty RL, Swensen SJ, Huprich JE, Thompson WG, Hoskin TL (2008). Feasibility of using a walking workstation during CT image interpretation. J Am Coll Radiol.

[67] Owen N (2012). Sedentary behavior: understanding and influencing adults’ prolonged sitting time. Prev Med.

[68] Gardner B, Smith L, Lorencatto F, Hamer M, Biddle SJ (2016). How to reduce sitting time? A review of behaviour change strategies used in sedentary behaviour reduction interventions among adults. Health Psychol Rev.

[69] Prince SA, Saunders TJ, Gresty K, Reid RD (2014). A comparison of the effectiveness of physical activity and sedentary behaviour interventions in reducing sedentary time in adults: a systematic review and meta-analysis of controlled trials. Obes Rev.

