# Supplementary material for: A systematic review and meta-analysis of the effect of treadmill desks on energy expenditure, sitting time and cardiometabolic health in adults
Source: BMC Public Health. 2021 Nov 13;21:2082. doi: 10.1186/s12889-021-12094-9 (PMC8590128; doi:10.1186/s12889-021-12094-9)
Supplement: Supplementary file 1 — Additional file 1: Table 1. Characteristics of the included studies in the laboratory settings. Description of data: study design, outcome of interest, estimated mean, and mean difference. Table 2. Characteristics of the included studies in the workplace settings. Description of data: study design, outcome of interest, estimated mean, and mean difference. Table 3. Treadmill desk in laboratory setting. Description of data: Effect estimates. Table 4. Treadmill desk in workplace setting. Description of data: Effect estimates [file 12889_2021_12094_MOESM1_ESM.zip › new BMC Public Health Additional File 1 - Table 3.docx]

| **Table 3. Treadmill desk in laboratory setting.** | | | | |
| --- | --- | --- | --- | --- |
| **Outcomes** | **No. of studies** | **Effect estimate (SE)** | **95% CI** | ***I*^2^ (%)** |
| ***Primary outcomes*** | |  |  |  |
| EE: kcal per hour | 4  [26,28-30] | 105.23  (7.56) | 90.41, 120.04 | 61.22 |
| ***Secondary outcomes*** | |  |  |  |
| VO_2_ (mL/kg/min) | 2  [28,30] | 5.0  (0.84) | 3.35, 6.64 | 86.19 |
|  |  |  |  |  |
| Systolic BP (mmHg) | 4  [27,28,31,32] | -1.26  (2.85) | -6.85, 4.33 | 45.57 |
|  |  |  |  |  |
| Diastolic BP (mmHg) | 4  [27,28,31,32] | -1.79  (1.68) | -5.08, 1.50 | 18.46 |
|  |  |  |  |  |
| Abbreviations: EE, energy expenditure; VO_2_, mass relative oxygen consumption; kcal, kilocalorie; BP, blood pressure. | | | | |
