# Supplementary material for: A systematic review and meta-analysis of the effect of treadmill desks on energy expenditure, sitting time and cardiometabolic health in adults
Source: BMC Public Health. 2021 Nov 13;21:2082. doi: 10.1186/s12889-021-12094-9 (PMC8590128; doi:10.1186/s12889-021-12094-9)
Supplement: Supplementary file 1 — Additional file 1: Table 1. Characteristics of the included studies in the laboratory settings. Description of data: study design, outcome of interest, estimated mean, and mean difference. Table 2. Characteristics of the included studies in the workplace settings. Description of data: study design, outcome of interest, estimated mean, and mean difference. Table 3. Treadmill desk in laboratory setting. Description of data: Effect estimates. Table 4. Treadmill desk in workplace setting. Description of data: Effect estimates [file 12889_2021_12094_MOESM1_ESM.zip › new BMC Public Health Additional File 1 - Table 4.docx]

| **Table 4. Treadmill desk in workplace setting.** | | | | |
| --- | --- | --- | --- | --- |
| **Outcomes** | **No. of studies** | **Effect estimate (SE)** | **95% CI** | ***I*^2^ (%)** |
| ***Primary outcomes*** | |  |  |  |
| Sitting time (mins per hour) during a full-day | 5  [24,33-36] | -1.73  (0.8) | -3.30, -0.17 | 42.4 |
| ***Exploratory analysis*** | |  |  |  |
| Sitting time (mins per hour) during working hours | 2  [33,36] | -3.58  (1.83) | -7.17, 0.02 | 0 |
| ***Secondary outcomes*** | |  |  |  |
| Systolic BP (mmHg) | 3  [33-35] | -3.83  (2.20) | -8.13, 0.48 | 0 |
|  |  |  |  |  |
| Diastolic BP (mmHg) | 3  [33-35] | -1.58  (1.91) | -5.32, 2.17 | 0 |
|  |  |  |  |  |
| Glucose (mg/dL) | 4  [33-35,37] | -0.48  (1.62) | -3.67, 2.70 | 0 |
|  |  |  |  |  |
| High density lipoprotein (mg/dL) | 3  [34,35,37] | 2.06  (2.43) | -2.70, 6.82 | 0 |
|  |  |  |  |  |
| Triglycerides (mg/dL) | 4  [33-35,37] | -12.69  (11.67) | -35.56, 10.17 | 0 |
|  |  |  |  |  |
| Cholesterol (mg/dL) | 3  [33-35] | -1.82  (5.58) | -12.76, 9.12 | 0 |
|  |  |  |  |  |
| Body fat (%) | 4  [34-37] | -0.28  (0.92) | -2.08, 1.52 | 0 |
|  |  |  |  |  |
| Body mass index (kg/m^2^) | 3  [24,33,36] | 0.37  (0.61) | -0.82, 1.56 | 0 |
