# Supplementary material for: A systematic review and meta-analysis of the effect of treadmill desks on energy expenditure, sitting time and cardiometabolic health in adults
Source: BMC Public Health. 2021 Nov 13;21:2082. doi: 10.1186/s12889-021-12094-9 (PMC8590128; doi:10.1186/s12889-021-12094-9)
Supplement: Supplementary file 1 — Additional file 1: Table 1. Characteristics of the included studies in the laboratory settings. Description of data: study design, outcome of interest, estimated mean, and mean difference. Table 2. Characteristics of the included studies in the workplace settings. Description of data: study design, outcome of interest, estimated mean, and mean difference. Table 3. Treadmill desk in laboratory setting. Description of data: Effect estimates. Table 4. Treadmill desk in workplace setting. Description of data: Effect estimates [file 12889_2021_12094_MOESM1_ESM.zip › Revision document - new BMC Public Health Additional File 1 - Table 1.docx]

| **Table 1. Characteristics of the included studies in the laboratory settings.** | | | | | | | |
| --- | --- | --- | --- | --- | --- | --- | --- |
| **Study** | **Population, sample size & location** | **Age, height, weight, or BMI: mean**  **(SD)** | **Design**  **& duration** | **Comparisons** | **Outcome of interest** | **Estimated**  **mean (SD)** | **Mean difference (SD)** |
| Levine & Miller, 2007 [29] | 15 (14 females)  healthy adults with high BMI and sedentary occupations.  Minnesota, United States | 43 (7.5) years; BMI: 32 (2.6) kg/m^2^ | Repeated-measures;  slow walking for 20 minutes; energy expenditure measured continuously throughout testing protocol using indirect calorimeter; time of measurement was not described | Treadmill desk (intervention, n=15: self-select walking speed ≤ 3 mph).  Office chair (control, n=15) | EE, energy expenditure | ***Energy expenditure:***  EE (intervention): 191 (29) kcal/h  EE (control): 72 (10) kcal/h | EE: 119 (7.9) kcal/h |
|  |  |  |  |  |  |  |  |
| Cox et al., 2011 [28] | 31 (22 females) healthy adults. Ohio,  United States | 37 (2.5) years; weight: 74.8 (16.2) kg | Random order testing; slow walking for 5 minutes; energy expenditure measured by open circuit spirometry; blood pressure measured with auscultation and sphygmomano-meter (time of measurement was not described); additional assessment of speech quality | Treadmill desk (intervention, n=31: walking speed ≤ 3.2 kmph).  Office chair (control, n=31) | EE, energy expenditure  Oxygen consumption(mass relative)  BP, blood pressure | ***Energy expenditure****:*  EE (intervention): 158.15 (39.32) kcal/h  EE (control): 68.39 (15.39) kcal/h  ***Oxygen consumption:***  VO_2_ (intervention), 7.4 (1.84) mL/kg/min  VO_2_ (control), 3.2 (0.72) mL/kg/min  ***Cardiometabolic risk factors:***  Systolic BP (intervention), 129 (16.7) mmHg  Systolic BP (control), 123 (16.7) mmHg  Diastolic BP (intervention), 80 (16.7) mmHg  diastolic BP (control), (76 (16.7) mmHg | EE: 89.76 (7.58) kcal/h  VO_2_: 4.2 (0.35) mL/kg/min (mass relative)  Systolic BP: 6.0 (4.24) mmHg  Diastolic BP: 4.0 (4.24) mmHg |
|  |  |  |  |  |  |  |  |
| Zeigler et al., 2015 [31] | 10 (6 females) prehyper-tensive adults; sedentary occupation. Arizona,  United States | 43 (13) years; BMI: 27(6) kg/m^2^ | Randomized crossover; slow walking (hourly) for 10 to 30 minutes in an 8-hour workday, 7 days apart; ambulatory blood pressure monitoring of non-dominant arm; measurements recorded every 15 minutes throughout the day (9:00 AM–10:00 PM), and every 60 minutes throughout the night (10:00 PM–6:00 AM) | Treadmill desk (intervention, n=10: walking speed ≤ 1 mph).  Seated at a desk (control, n=10). | Blood pressure | ***Cardiometabolic risk factors:***  Systolic BP (intervention), 134 (14) mmHg  Systolic BP (control), 137 (16) mmHg  Diastolic BP (intervention), 79 (10) mmHg  Diastolic BP (control), 82 (12) mmHg | Systolic BP:  -3.0 (6.72) mmHg  Diastolic BP:  -3.0 (4.94) mmHg |
|  |  |  |  |  |  |  |  |
| Zeigler et al., 2016 [32] | 9 (7 females) adults with high BMI, prehyper-tension or impaired FG, and sedentary occupations. Arizona,  United States | 30 (15) years; BMI: 28.7 (2.7) kg/m^2^ | Randomized crossover full-factorial; slow walking (hourly) for 10 to 30 minutes in an 8-hour workday, 7 days apart; ambulatory blood pressure monitoring of non-dominant arm; measurements recorded every 15 minutes throughout the day (9:00 AM–10:00 PM) | Treadmill desk (intervention, n=9: walking speed ≤ 1 mph).  Seated at a desk (control, n=9). | Blood pressure | ***Cardiometabolic risk factors:***  Systolic BP (intervention), 131 (16) mmHg  Systolic BP (control), 134 (17) mmHg  Diastolic BP (intervention), 73 (11) mmHg  Diastolic BP (control), 72 (15) mmHg | Systolic BP:  -3.0 (7.8) mmHg  Diastolic BP: 1.0 (5.43) mmHg |
|  |  |  |  |  |  |  |  |
| Botter et al., 2016 [26] | 12 (6 females) healthy adults with sedentary occupations. Sankt Augustin, Germany | 38.7 (11.4) years; height: 171 (9) cm, weight: 75 (15.4) kg) | Randomised repeated-measures; slow walking for ~20 minutes; energy expenditure measured by heart rate and indirect calorimeter in the morning or afternoon as assigned | Treadmill desk (intervention, n=12: walking speed 0.6 and 2.5 kmph).  Office chair (control, n=12). | EE, energy expenditure | ***Energy expenditure in kcals per hour:***  EE (intervention) at 2.5 kmph, 210 (45) kcal/h  EE (control) at rest, 112.5 kcal/h  ***Metabolic Equivalent of Task:***  METs (intervention) at 2.5 kmph, 2.8 (0.6)  METs (control) at rest, 1.5 | EE: 97.5 (13) kcal/h |
|  |  |  |  |  |  |  |  |
| Champion et al., 2018 [27] | 24 (12 females) healthy adults with sedentary occupations; Bedfordshire, United Kingdom | 35.8 (10.4) years; height: 171.5 (5.3) cm, weight: 76.1 (16.1) kg | Randomised crossover; slow walking (hourly) for 20 minutes, in a 6.5-hour workday; resting brachial blood pressure measurement of the left arm in seated upright position using an automatic device in the morning (08:30); three measurements of baseline blood pressure with a 2-minute rest in between each reading; single measurements recorded at 60, 120, 180, 240, 300, 360, and 390 minutes | Treadmill desk (intervention, n=24: self-select walking speed ≤ 3.5 kmph).  Seated at a desk (control, n=24). | Blood pressure | ***Cardiometabolic risk factors:***  Systolic BP (intervention), 118 (3.55) mmHg  Systolic BP (control), 122 (4.74) mmHg  Diastolic BP (intervention), 74 (3.55) mmHg  Diastolic BP (control), 77 (3.55) mmHg | Systolic BP:  -4.0 (1.21) mmHg  Diastolic BP:  -3.0 (1.02) mmHg |
|  |  |  |  |  |  |  |  |
| Schuna et al., 2019 [30] | 16 (8 females) healthy adults with sedentary occupations; Louisiana, United States | 33.9 (7.1) years; height: 172.5 (6) cm,  weight: 67.4 (7.9) kg | Repeated-measures; slow walking for 15 minutes; energy expenditure measured continuously throughout testing protocol using indirect calorimeter | Treadmill desk (intervention, n=16: self-select walking speed ≤ 3.2 kmph).  Office chair (control, n=16). | EE, energy expenditure  Oxygen consumption(mass relative) | ***Energy expenditure:***  EE (intervention): 3.07 (0.66) kcal/min  EE (control): 1.17 (0.21) kcal/min  ***Oxygen consumption:***  VO_2_ (intervention), 9.52 (1.92) mL/kg/min  VO_2_ (control), 3.64 (0.73) mL/kg/min | EE: 114 (10.3) kcal/h  VO_2_: 5.9 (0.51) mL/kg/min (mass relative) |
|  |  |  |  |  |  |  |  |
| Abbreviations: BMI, body mass index; mph, miles per hour; kmph, kilometers per hour; kcal, kilocalories. EE, energy expenditure; BP, blood pressure; MET, metabolic equivalent of task | | | | | | | |
