# Supplementary material for: A systematic review and meta-analysis of the effect of treadmill desks on energy expenditure, sitting time and cardiometabolic health in adults
Source: BMC Public Health. 2021 Nov 13;21:2082. doi: 10.1186/s12889-021-12094-9 (PMC8590128; doi:10.1186/s12889-021-12094-9)
Supplement: Supplementary file 1 — Additional file 1: Table 1. Characteristics of the included studies in the laboratory settings. Description of data: study design, outcome of interest, estimated mean, and mean difference. Table 2. Characteristics of the included studies in the workplace settings. Description of data: study design, outcome of interest, estimated mean, and mean difference. Table 3. Treadmill desk in laboratory setting. Description of data: Effect estimates. Table 4. Treadmill desk in workplace setting. Description of data: Effect estimates [file 12889_2021_12094_MOESM1_ESM.zip › Revision document - new BMC Public Health Additional File 1 - Table 2.docx]

| **Table 2. Characteristics of the included studies in the workplace settings.** | | | | | | | |
| --- | --- | --- | --- | --- | --- | --- | --- |
| **Study** | **Population, sample size & location** | **Age, height, weight, or BMI: mean**  **(SD)** | **Design**  **& duration** | **Comparisons** | **Outcome of interest** | **Estimated**  **mean (SD)** | **Mean difference (SD)** |
| John et al., 2011 [34] | 12 (7 females) adults with high BMI and sedentary occupations. Tennessee, United States | 46.2 (9.2) years;  BMI: 33.9 (5) kg/m^2^ | Pre-post study with follow-up at 3 and 9 months; accelerometer-derived sitting time (thigh-placement device worn during all waking hours for 2 workdays prior to laboratory visits); body fat measured by air displacement plethysmography and dual energy x-ray absorptiometry; blood drawn after 12-hour fast to ascertain lipid and metabolic profiles; seated resting blood pressure measured using auscultation; time of measurement was not described | Treadmill desk (intervention: self-select speed ~2 mph). Office chair (control) | ST, sitting time (during unspecified waking hours)  BP, blood pressure  FG, fasting glucose  HDL, high density lipoprotein  TG, triglycerides  TC, total cholesterol  BF, body fat | After a 9-month follow-up  ***Sitting time during a full-day:***  ST at baseline: 1235 (79) min/day, or 51.46 (3.29) min/h  ST at 9 months: 1138 (112) min/day, or 47.42 (4.67) min/h  ***Cardiometabolic risk factors:***  Systolic BP at baseline: 125 (11) mmHg  Systolic BP at 9 months: 118 (9) mmHg  Diastolic BP at baseline: 77 (8) mmHg  Diastolic BP at 9 months: 79 (9) mmHg  FG at baseline: 95 (6) mg/dL  FG at 9 months: 94 (6) mg/dL  HDL at baseline: 47 (11) mg/dL  HDL at 9 months: 50 (8) mg/dL  TG at baseline: 177 (68) mg/dL  TG at 9 months: 152 (54) mg/dL  TC at baseline: 206 (31) mg/dL  TC at 9 months: 195 (36) mg/dL  BF at baseline: 44.4 (5.8) percent  BF at 9 months: 42.4 (7.4) percent | Sitting time during a full-day: -4.04 (1.65) min/h  Systolic BP: -7.0 (4.1) mmHg  Diastolic BP: 2.0 (3.5) mmHg  FG: -1.0 (2.5) mg/dL  HDL: 3 (3.9) mg/dL  TG: -25 (25.1) mg/dL  TC: -11 (13.7) mg/dL  BF (%): -2.0 (2.7) |
|  |  |  |  |  |  |  |  |
| Koepp et al., 2013 [35] | 36 (25 females) adults with sedentary occupations. Minnesota, United States | 42 (9.9) years;  BMI: 29 (7) kg/m^2^ | Prospective clinical trial with follow-up at 6 and 12 months; accelerometer-derived sitting time (hip-placement device worn during waking hours, 7 days a week, for 12 months); body fat measured by air displacement plethysmography; energy expenditure measured by indirect calorimeter; blood drawn to ascertain lipid and metabolic profiles; automated cuff was used to obtain duplicates of resting (30 minutes of rest) blood pressure; time of measurement was not described | Treadmill desk (intervention: self-select speed ≤ 2 mph).  Office chair (control) | ST, sitting time (during unspecified waking hours)  BP, blood pressure  FG, fasting glucose  HDL, high density lipoproteins  TG, triglycerides  TC, total cholesterol  BF, body fat | After a 12-month follow-up  ***Sitting time during a full-day:***  ST at baseline: 1020 (75) min/day, or 42.5 (3.13) min/h  ST at 12 months: 978 (95) min/day, or 40.75 (3.96) min/h  ***Cardiometabolic risk factors:***  Systolic BP at baseline: 132 (13) mmHg  Systolic BP at 12 months: 129 (13) mmHg  Diastolic BP at baseline: 89 (18) mmHg  Diastolic BP at 12 months: 84 (9) mmHg  FG at baseline: 93.8 (23) mg/dL  FG at 12 months: 92.7 (11.1) mg/dL  HDL at baseline: 55 (20) mg/dL  HDL at 12 months: 59 (23) mg/dL  TG at baseline: 133 (88) mg/dL  TG at 12 months: 121 (57) mg/dL  TC at baseline: 193 (32) mg/dL  TC at 12 months: 193 (28) mg/dL  BF at baseline: 31.4 (7.9) percent  BF at 12 months: 30.5 (8.6) percent | Sitting time during a full-day: -1.75 (0.84) min/h  Systolic BP: -3.0 (3.1) mmHg  Diastolic BP: -5.0 (3.4) mmHg  FG: -1.1 (4.3) mg/dL  HDL: 4.0 (5.1) mg/dL  TG: -12 (17.5) mg/dL  TC: 0.0 (7.1) mg/dL  BF (%): -0.9 (1.95) |
|  |  |  |  |  |  |  |  |
| Schuna et al., 2014 [36] | 41 (40 females) adults (31 included in analysis) with high BMI and sedentary occupations. Louisiana, United States | 40.1 (10.1) years;  BMI: 35.8 (8.3) kg/m^2^ | Randomised controlled trial with follow-up at 3 months; accelerometer-derived sitting time (hip-placement device was worn for 24 hours/day for at least 4 working days (weekdays) at baseline and follow-up); body fat measured by bioelectrical impedance; time of measurement was not described | Shared treadmill desk (intervention, n=15: self-select walking speed ≤ 2 mph and received behavioural support reminders). Office chair (control, n=16) | ST, sitting time (during ~20 hours of time spent awake in a full-day, and ~8.2 working hours)  BF, body fat  BMI | After a 3-month follow-up  ***Sitting time during a full-day:***  ST (intervention): 41.3 (3.5) min/h  ST (control): 44.3 (4.3) min/h  ***Sitting time*** ***during working hours:***  ST (intervention): 38.4 (5.8) min/h  ST (control): 42.8 (6.4) min/h  ***Cardiometabolic risk factors:***  BF (intervention): 44.9 (5.4) percent  BF (control): 42.8 (6.8) percent  BMI (intervention): 36.6 (8.9) kg/m^2^  BMI (control): 36.8 (9.2) kg/m^2^ | Sitting time during a full-day: -3 (1.4) min/h  Sitting time during working hours: -4.4 (2.19) min/h  BF (%): 2.1 (2.2)  BMI: -0.2 (3.3) kg/m^2^ |
|  |  |  |  |  |  |  |  |
| Thompson et al., 2014 [37] | 20 (3 females) physicians (17 included in analysis) with high BMI. Minnesota, United States | 47.9 (7.7) years; weight: 53.8 (7.5) kg | Randomized crossover trial with follow-up at 2, 14, and 26 weeks; body fat measured by dual energy x-ray absorptiometry; accelerometer-derived energy expenditure via hip-placement device worn for the duration of study; fasting blood chemistry (lipid and metabolic profiles); time of measurement was not described | Treadmill desk (intervention first, n=8: self-select walking speed 1 mph). Office chair (control first, n=9). All participants received exercise counselling | FG, fasting glucose  HDL, high density lipoproteins  TG, triglycerides  BF, body fat | After a 3-month follow-up  ***Cardiometabolic risk factors among treadmill desk users:***  Change in FG: -4 (10.44) mg/dL  Change in HDL: 0 (3.89) mg/dL  Change in TG: 9.82 (48.89) mg/dL  Change in BF: -0.44 (1.31) percent |  |
|  |  |  |  |  |  |  |  |
| Bergman et al., 2018 [33] | 80 (44 females) adults (79 included in analysis) with high BMI and sedentary occupations. Umeå,  Sweden | 51.4 (6.8) years;  BMI: 25–40 kg/m^2^ | Randomised controlled trial with follow-up at 2, 6, 10, and 13 months; accelerometer-derived sitting time (thigh-placement device was worn for 24 hours a day, 7 consecutive days, and a hip-placement device was worn during all waking hours for 14 consecutive days at each follow-up, in order to measure total daily walking time (at weekdays and weekends), and intensity of physical activity, respectively); overnight fasting blood chemistry (lipid and metabolic profiles); time of measurement was not described | Treadmill desk (intervention, n=39: self-select speed < 5 mph). Sit-stand desk (control, n=40). Both groups received behavioural support (health consultation, emailed reminders, and health-promotion programs, such as subsidized gym fees and 1-hour per week gym leave) | ST, sitting time (during ~16.5 hours of time spent awake in a full-day, and ~8.84 working hours)  BP, blood pressure  FG, fasting glucose  TG, triglyceride  TC, total cholesterol  BMI | After a 13-month follow-up  ***Sitting time during waking hours:***  ST (intervention): 555 (173) minutes, or 33.59 (10.46) min/h  ST (control): 565 (170) minutes, or 34.45 (10.39) min/h  ***Sitting time*** ***during working hours:***  ST (intervention): 271 (133) minutes, or 30.62 (14.99) min/h  ST (control): 285 (130) minutes, or 32.28 (14.7) min/h  ***Cardiometabolic risk factors:***  Systolic BP (intervention): 129.1 (26.6) mmHg  Systolic BP (control): 130.5 (14.3) mmHg  Diastolic BP (intervention): 81.6 (17.2) mmHg  Diastolic BP (control): 83.1 (8.7) mmHg  FG (intervention): 103.2 (13.9) mg/dL  FG (control): 102.7 (8.3) mg/dL  TG (intervention): 115.1 (26.6) mg/dL  TG (control): 124 (100.1) mg/dL  TC (intervention): 205 (56.1) mg/dL  TC (control): 205 (48.7) mg/dL  BMI (intervention): 29.8 (4.1) kg/m^2^  BMI (control): 29 (4.2) kg/m^2^ | Sitting time during a full-day: -0.86 (2.35) min/h  Sitting time during working hours:  -1.66 (3.34) min/h  Systolic BP: -1.4 (4.9) mmHg  Diastolic BP: -1.5 (3.1) mmHg  FG: 0.5 (2.6) mg/dL  TG: -8.9 (22.0) mg/dL  TC: 0.0 (12.1) mg/dL  BMI: 0.8 (0.94) kg/m^2^ |
|  |  |  |  |  |  |  |  |
| Wahlström et al., 2019 [24] | 86 (74 females) adults (59 included in analysis) with sedentary occupations. Örnsköldsvik, Sweden | 48.4 (10.4) years; BMI: 25.7 (4.14) kg/m^2^ | prospective controlled design with follow-up at 6, 11, and 18 months; accelerometer-derived sitting time (thigh-placement device was worn for 24 hours a day, and a hip-placement device was worn during waking hours) | Shared treadmill desk in a flex office (intervention, n=29). Sit-stand desk in a traditional cell office (control, n=30). Both groups received multi-component behavioural support (health workshops, communiqués, automated reminders, and health-promotion programs, such as subsidized gym fees and 1-hour per week gym leave) | ST, sitting time (during ~16 hours of time spent awake in a full-day, and ~8 working hours)  BMI | After an 18-month follow-up  ***Sitting time during waking hours:***  ST (intervention): 531 (70.98) minutes, or 33.19 (4.44) min/h  ST (control): 522 (74.99) minutes, or 32.63 (4.69) min/h  ***Sitting time*** ***during working hours:***  ST (intervention): 258 (61.78) minutes, or 32.25 (7.72) min/h  ST (control): 250 (64.27) minutes, or 31.25 (8.03) min/h  ***Cardiometabolic risk factors:***  BMI (intervention): 26.2 (3.75) kg/m^2^  BMI (control): 24.6 (3.55) kg/m^2^ | Sitting time during a full-day: 0.56 (1.19) min/h  Sitting time during working hours:  1.0 (2.05) min/h  BMI: 1.6 (2.05) kg/m^2^ |
|  |  |  |  |  |  |  |  |
| Abbreviations: BMI, body mass index; mph, miles per hour; kmph, kilometers per hour; BP, blood pressure; FG, fasting glucose; HDL, high density lipoprotein; TG, triglycerides; TC, total cholesterol; BF, body fat | | | | | | | |
