# Supplementary material for: A systematic review and meta-analysis of the effect of treadmill desks on energy expenditure, sitting time and cardiometabolic health in adults
Source: BMC Public Health. 2021 Nov 13;21:2082. doi: 10.1186/s12889-021-12094-9 (PMC8590128; doi:10.1186/s12889-021-12094-9)
Supplement: Supplementary file 2 — Additional file 2: Figure 1. Flowchart of studies included in the meta-analysis. Description of data: Flowchart of studies included in the meta-analysis. Figure 2. Forest plots of studies in laboratory settings. Description of data: Outcomes are as follows: (a) energy expenditure; (b) rate of oxygen consumption; (c) systolic blood pressure; (d) diastolic blood pressure. Error bars represent the 95% Confidence Interval. Data source: Botter et al., 2016 [26]; Champion et al., 2018 [27]; Cox et al., 2011 [28]; Levine & Miller, 2007 [29]; Schuna et al., 2019 [30]; Zeigler et al., 2015 [31]; Zeigler et al., 2016 [32]. Figure 3. Forest plots of studies in workplace settings. Description of data: Outcomes are as follows: (a) sitting time (minutes per hour) during a full-day; (b) sitting time (minutes per hour) during working hours; (c) systolic blood pressure; (d) diastolic blood pressure; (e) glucose concentrations; (f) high density lipoproteins: (g) triglycerides; (h) total cholesterol; (i) body fat percentage; (j) BMI. Error bars represent the 95% Confidence Interval. Data source: Wahlström et al., 2019 [24]; Bergman et al., 2018 [33]; John et al., 2011 [34]; Koepp et al., 2013 [35]; Schuna et al., 2014 [36]; Thompson et al., 2014 [37]. Figure 4. Funnel plots of studies in laboratory settings. Description of data: Outcomes are as follows: (a) energy expenditure; (b) rate of oxygen consumption; (c) systolic blood pressure; (d) diastolic blood pressure. Error bars represent the 95% Confidence Interval. Data source: Botter et al., 2016 [26]; Champion et al., 2018 [27]; Cox et al., 2011 [28]; Levine & Miller, 2007 [29]; Schuna et al., 2019 [30]; Zeigler et al., 2015 [31]; Zeigler et al., 2016 [32]. Figure 5. Funnel plots of studies in workplace settings. Description of data: Outcomes are as follows: (a) sitting time (minutes per hour) during a full-day; (b) sitting time (minutes per hour) during working hours; (c) systolic blood pressure; (d) diastolic blood pressure; (e) g [file 12889_2021_12094_MOESM2_ESM.zip › new BMC Public Health Additional File 2 - Figure 4.docx]

| A | B |
| --- | --- |
| 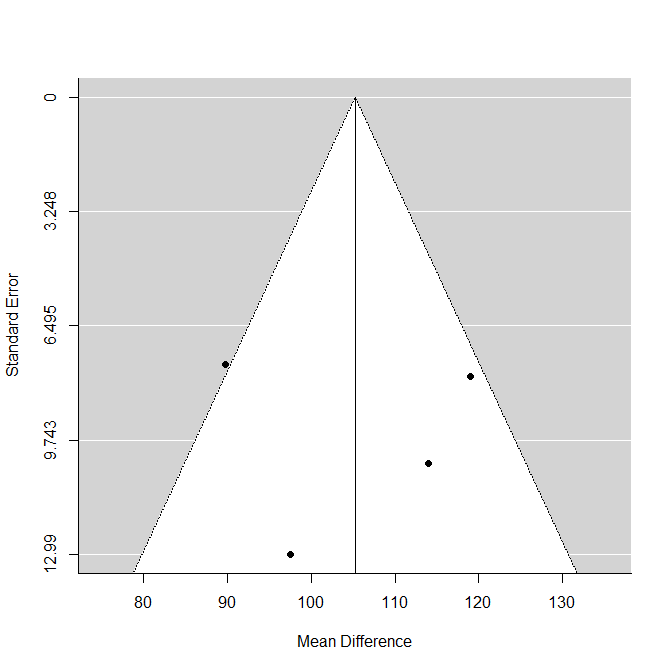 | 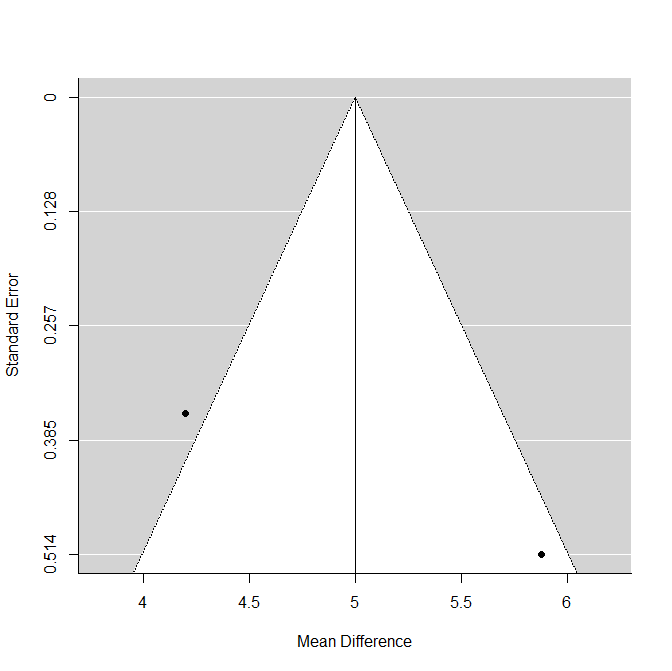 |
| C | D |
| 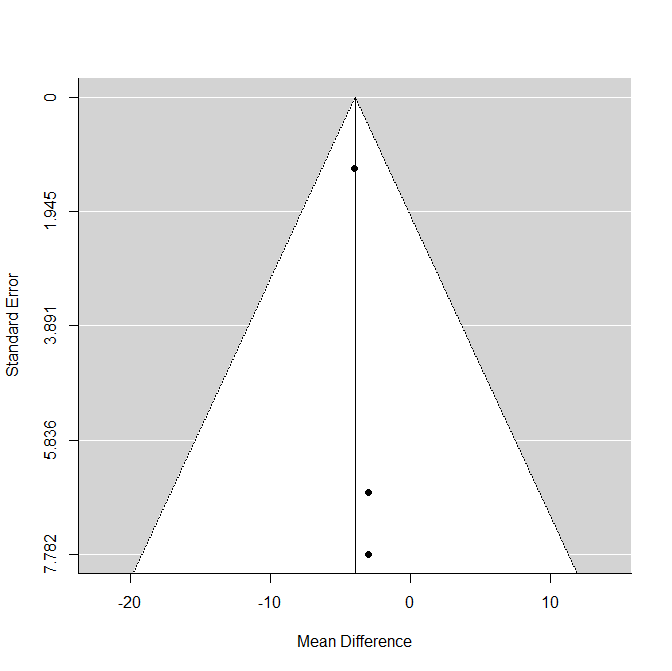 | 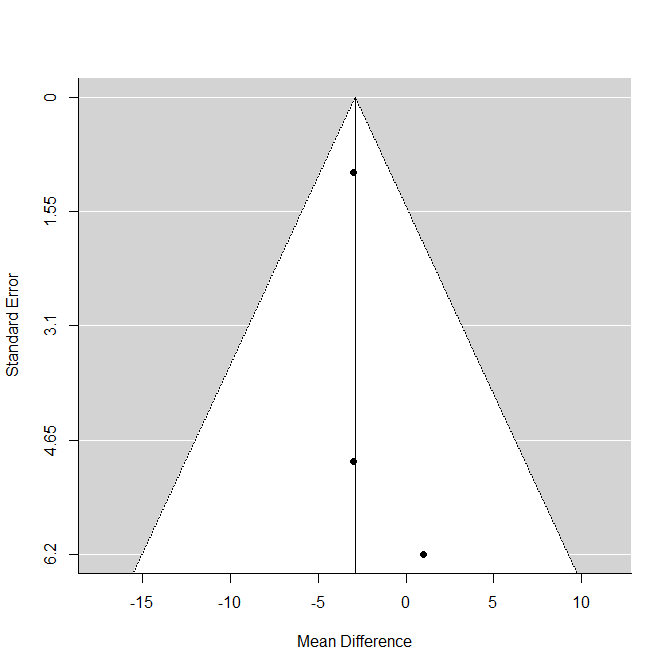 |
|  |  |
| **Figure 4.** Funnel plots of studies in laboratory settings. Outcomes are as follows: (a) energy expenditure; (b) rate of oxygen consumption relative to body mass; (c) systolic blood pressure; (d) diastolic blood pressure. Error bars represent the 95% Confidence Interval. | |
