# Supplementary material for: A systematic review and meta-analysis of the effect of treadmill desks on energy expenditure, sitting time and cardiometabolic health in adults
Source: BMC Public Health. 2021 Nov 13;21:2082. doi: 10.1186/s12889-021-12094-9 (PMC8590128; doi:10.1186/s12889-021-12094-9)
Supplement: Supplementary file 3 — Additional file 3: Table S1. Electronic search strategy [file 12889_2021_12094_MOESM3_ESM.docx]

**A systematic review and meta-analysis of the effect of treadmill desks on energy expenditure, sitting time and cardiometabolic health in adults**

**Authors**

**Akinkunle Oye-Ṣomẹfun**

222A Bethune College, York University, 4700 Keele Street, Toronto, ON, M3J 1P3, Canada.

akinoyes@yorku.ca

**Zahra Azizi**

McGill University Health Centre Research Institute, Centre for Outcomes Research and Evaluation (CORE), 5252 De Maisonneuve Blvd, Montréal, QC H4A 3S5, Canada.

zahra.azizimd@gmail.com

**Chris I. Ardern**

344 Bethune College, York University, 4700 Keele Street, Toronto, ON, M3J 1P3, Canada. cardern@yorku.ca

**Michael Rotondi**

364 Bethune College, York University, 4700 Keele Street, Toronto, ON, M3J 1P3, Canada.

mrotondi@yorku.ca

**Address for Correspondence:**

Akinkunle Oye-Ṣomẹfun

222A Bethune College

York University

4700 Keele Street, Toronto, ON

M3J1P3

Email: akinoyes@yorku.ca

| **Table S1:** Electronic search strategy | |
| --- | --- |
| Database | Searchfield |
| PubMed | ((("Workplace"[Mesh]) OR ((((work* OR job*))) AND ((plac* OR locat* OR sit* OR offic*))))) AND (("Walking"[Mesh]) OR ((((treadmil* OR walk*))) AND ((desk* OR (work* AND station*) OR (work* AND plac*) OR offic* OR devic* OR interven* OR (work* AND set*))))) |
